# Risk factors for vancomycin resistance in patients with *Enterococcus faecium* bloodstream infections: an analysis of the Munich Multicentric Enterococci Cohort

**DOI:** 10.1128/spectrum.00052-25

**Published:** 2025-06-05

**Authors:** Laura Wagner, Milena Wurst, Johanna Erber, Tobias Bachfischer, Bernhard Haller, Sabine Gleich, Karl Dichtl, Roland M. Schmid, Julian Triebelhorn, Florian Voit, Ulrich Seybold, Dirk H. Busch, Patrick C. Rämer, Christoph D. Spinner, Jochen Schneider, Kathrin Rothe

**Affiliations:** 1 Department of Internal Medicine II, TUM School of Medicine and Health, TUM University Hospital, Technical University of Munich 9184, Munich, Germany; 2 German Centre for Infection Research (DZIF), partner site Munich, Munich, Germany; 3 Institute of AI and Informatics in Medicine, TUM School of Medicine and Health, Technical University of Munich, Munich, Germany; 4 Public Health Service, City of Munich, Munich, Germany; 5 Max von Pettenkofer Institute for Hygiene und Medical Microbiology, Faculty of Medicine, Ludwig Maximilian University of Munich 54187, Munich, Germany; 6 Diagnostic and Research Institute of Hygiene, Microbiology and Environmental Medicine, Medical University of Graz 31475, Graz, Austria; 7 Division of Infectious Diseases, Department of Medicine IV, Hospital of the Ludwig Maximilian University of Munich, Munich, Germany; 8 Institute for Medical Microbiology, Immunology and Hygiene, TUM School of Medicine and Health, Technical University of Munich, Munich, Germany; 9 Department of Hospital Hygiene and Infection Control, Munich Municipal Hospital Group 9225, Munich, Germany; MultiCare Health System, Tacoma, Washington, USA

**Keywords:** vancomycin resistance, *Enterococcus*, bloodstream infections, *Enterococcus faecium*, antibiotic resistance

## Abstract

*Enterococcus faecium* is difficult to treat owing to its intrinsic and acquired resistance to antibiotics, particularly vancomycin. Vancomycin-resistant *Enterococcus faecium* is an important cause of bloodstream infections in healthcare settings with limited treatment options. This study aimed to analyze the risk factors for vancomycin-resistant *Enterococcus faecium* bloodstream infection. This retrospective analysis of data from the Munich Multicentric Enterococci Cohort analyzed 200 episodes of nonrecurrent vancomycin-susceptible *Enterococcus faecium* bloodstream infection and 196 episodes of nonrecurrent vancomycin-resistant *Enterococcus faecium* bloodstream infection from six hospitals in Munich, Germany, between 2010 and 2019. Logistic regression was used to identify risk factors for vancomycin-resistant *Enterococcus faecium* bloodstream infection. In the unadjusted analysis, the risk factors for vancomycin-resistant *Enterococcus faecium* bloodstream infection included the length of hospital stay, previous treatment with vancomycin or linezolid, and solid organ transplantation. In the multivariable analysis, prior treatment with vancomycin and solid organ transplantation were independent risk factors for vancomycin-resistant *Enterococcus faecium* bloodstream infection. Vancomycin resistance was not significantly associated with the severity of underlying diseases. This study identified prior vancomycin treatment and solid organ transplantation as key independent risk factors for vancomycin-resistant *Enterococcus faecium* bloodstream infection.

**IMPORTANCE:**

Vancomycin-resistant *Enterococcus faecium* is a growing threat in healthcare settings, challenging the management of enterococcal infections. This study identified prior treatment with vancomycin and solid organ transplantation as risk factors for vancomycin-resistant *Enterococcus faecium* bloodstream infections. Based on the analysis of five disease severity scores for acute and chronic illness, the severity of underlying diseases could not be demonstrated to be a risk factor for the occurrence of vancomycin resistance in *Enterococcus faecium* bloodstream infections.

## INTRODUCTION

Enterococcal infections pose a substantial clinical and therapeutical challenge owing to intrinsic and acquired antibiotic resistance (1). *Enterococcus faecium* (ECFM) is a major cause of bloodstream infections (BSI) and the third most common hospital-acquired pathogen in the United States (2). Treatment options are limited, owing to the increasing prevalence of vancomycin-resistant *Enterococcus faecium* (VRE). In 2021, antimicrobial susceptibility testing results for vancomycin were available for 98.6% of all reported invasive ECFM isolates in Europe. The national prevalences of VRE in European countries ranged from 0.0% to 66.4% of the total number of invasive ECFM isolates tested, with increasing rates between 2017 and 2021 (1).

Studies have highlighted the potential risk factors for VRE BSI, including prolonged hospital stay, intensive care unit (ICU) admission, and prior antibiotic exposure, especially to vancomycin and other broad-spectrum antibiotics, such as cephalosporins, central venous catheter use, and known colonization with VRE. Additionally, underlying comorbidities, such as acute and chronic renal disease, diabetes, hematological malignancies, and immunosuppression, are associated with an increased risk of VRE BSI (3–9).

Enterococcal BSIs are associated with high mortality if not adequately treated (10). Vancomycin is typically used for empiric treatment of ECFM BSI. However, in the case of vancomycin resistance, susceptibility testing, which usually takes 24 to 48 hours, is required for effective treatment. In patients with BSI, inadequate antibiotic treatment is associated with increased mortality (11). Therefore, it is important to identify the risk factors for vancomycin resistance in patients with ECFM BSI to provide treating physicians with guidance on how to assess the chances for the causative ECFM to be resistant to vancomycin. This is necessary to enable adequate treatment to be initiated at the time of ECFM BSI diagnosis, before the susceptibility testing results are available. In this study, we aimed to identify risk factors for VRE BSI using demographical and clinical data from the Munich Multicentric Enterococci Cohort.

## MATERIALS AND METHODS

### Study design

**Fig 1:**
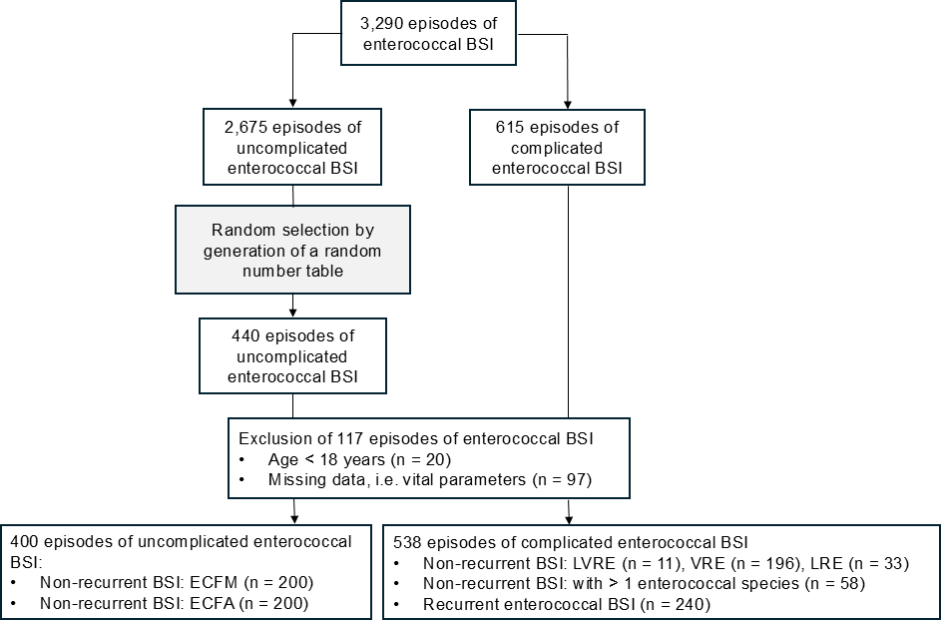
Flow chart of study cohort selection. BSI, bloodstream infection; ECFA, *Enterococcus faecalis*; ECFM, *Enterococcus faecium*; LRE, linezolid-resistant *Enterococcus faecium*; LVRE, linezolid- and vancomycin-resistant *Enterococcus faecium*; VRE, vancomycin-resistant *Enterococcus faecium*. Adapted from (12).

Clinical data from the Munich Multicentric Enterococci Cohort were analyzed retrospectively (12). This cohort includes patients aged ≥18 years from six hospitals in Munich, Germany (four centers of the Munich Municipal Hospital, the University Medical Center of the Technical University of Munich, and the University Hospital of the Ludwig Maximilian University of Munich) between 2010 and 2019. It comprises 2,675 uncomplicated and 615 complicated episodes of enterococcal BSI. Because of the large sample size of patients with uncomplicated enterococcal BSI, cases were selected using a random number table. In total, 440 episodes of uncomplicated enterococcal BSI and 615 episodes of complicated enterococcal BSI were identified. After excluding 117 episodes in patients aged <18 years (*n* = 20) and with missing data (*n* = 97), 200 episodes of nonrecurrent ECFM BSI and 196 episodes of nonrecurrent VRE BSI were identified and included in the analysis. (Fig. 1)

Complicated enterococcal BSI was defined as meeting any of the following criteria: (a) a positive blood culture (BC) with linezolid-resistant enterococci, VRE, or linezolid- and VRE; (b) BSI involving two different enterococcal species within the same episode; or (c) recurrent enterococcal BSI with more than one episode within 30 days. A separate enterococcal BSI episode was identified if another positive BC for *Enterococcus* species occurred >30 days after the initial positive BC. Uncomplicated enterococcal BSI was defined as BSI caused by *Enterococcus faecalis* or ECFM susceptible to vancomycin and linezolid that did not meet the criteria for complicated enterococcal BSI.

### Sample collection and selection

Data were extracted from electronic medical records and laboratory information management systems using HyBase software (epiNet AG, Bochum, Germany). Variables analyzed included the Pitt Bacteremia Score (PBS), Charlson Comorbidity Index (CCI), Sequential Organ Failure Assessment (SOFA) score, Acute Physiology And Chronic Health Evaluation (APACHE II) score, Simplified Acute Physiology Score (SAPS) II, the most relevant sources of infection (neutropenic fever, intra-abdominal, foreign body (central venous catheter including venous port system, dialysis shunt), endocarditis, urogenital), length of hospital stay prior to BSI, classification of BSI as community-acquired or nosocomial, hospital stay in the previous three months, route of admission to the hospital (from home, nursing home, hospital, rehabilitation centre), comorbidities (chronic dialysis requirement, solid organ transplantation, bone marrow transplantation, liver cirrhosis, tumour metastases), and age ≥65 years. All antibiotic treatments administered during the same hospital stay as the BSI were recorded. Data were collected on the classes of antibiotics, the start of treatment, and the duration of treatment. Antibiotic treatment was considered in the unadjusted and multivariable logistic regression analyses if initiated before the BSI diagnosis. The results of rectal screening for VRE were included if they were performed during the same hospital stay as the BSI. VRE was diagnosed by cultivation on chromogenic agar and automated antimicrobial susceptibility testing for rectal screening. Polymerase chain reaction (PCR) was additionally included initially in selected cases as a rapid screening measure. No molecular data on VRE was included.

### Statistical analysis

Variables with more than 33% missing values were excluded from the data set prior to the analysis. These variables included the APACHE II score and prior rectal VRE colonization. For the remaining variables with missing values, the MissForest algorithm, a random forest-based imputation method, was used to impute the missing values.

Quantitative data are summarized by mean and standard deviation (SD), and categorical data are reported as absolute and relative frequencies. Group comparisons of categorical variables were performed using Fisher’s exact test or Pearson’s chi-squared test, whereas continuous variables were compared using the Wilcoxon rank-sum test.

After imputing missing values, unadjusted and multivariable logistic regression analyses were conducted to identify factors associated with VRE BSI. Odds ratios (OR) with 95% confidence intervals (CI) were calculated for both the unadjusted and multivariable analyses to quantify the strength of the association between the predictor variables and VRE BSI.

Statistical hypothesis testing was performed using two-sided *P* values with the threshold for statistical significance set at 0.05. All statistical analyses were performed using R version 4.0.3 (R Foundation for Statistical Computing, Vienna, Austria).

## RESULTS

### Baseline characteristics

**Fig 2:**
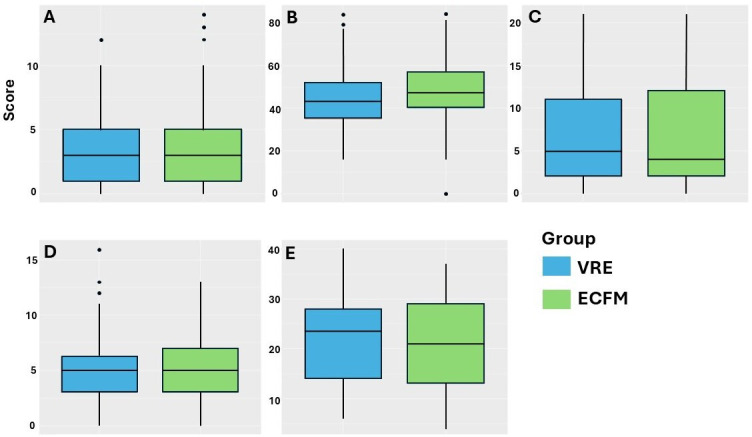
Box and whisker plots of the disease severity scores in patients with vancomycin-resistant enterococcus (VRE) bloodstream infection (BSI) (blue boxes) and *Enterococcus faecium* (ECFM) BSI (green boxes). (**A**) Pitt Bacteremia Score (PBS); (**B**) Simplified Acute Physiology Score (SAPS); (**C**) Sequential Organ Failure Assessment (SOFA) score (**D**) Charlson Comorbidity Index (CCI); and (**E**) Acute Physiology and Chronic Health Evaluation (APACHE II).

A total of 196 episodes of nonrecurrent VRE BSI and 200 episodes of nonrecurrent ECFM BSI were included. The mean (SD) of the CCI was 5.3 (3.0), APACHE II score was 21.6 (8.6), SOFA score was 6.8 (5.9), SAPS score was 45.9 (14.5), and PBS was 3.3 (2.9). None of the severity scores differed significantly between episodes with VRE BSI and those with ECFM BSI (Fig. 2).

The most common sources of infection were foreign bodies (central venous catheters, dialysis shunts) colonized with Enterococci (78 episodes, 19.7%), neutropenic fever (46 episodes, 11.6%), endocarditis (10 episodes, 2.5%), intra-abdominal focus (four episodes, 1.0%), and urogenital focus (three episodes, 0.8%), with no significant difference between episodes with VRE BSI and those with ECFM BSI.

Previous solid organ transplantation was more common in VRE BSI episodes (*n* = 24, 12.2%) than in ECFM BSI episodes (*n* = 12, 6.0%; *P* = 0.031). No significant differences were found in patients with chronic dialysis, bone marrow transplantation, liver cirrhosis, hematological malignancy, or metastatic cancer in the VRE BSI and ECFM BSI groups.

Out of all VRE and ECFM BSI episodes, a screening for rectal VRE was available before the diagnosis of BSI in 101 cases (58.7%). Patients with VRE BSI were significantly more likely to be screened positive for rectal VRE during the same hospital stay as patients with ECFM BSI (VRE, 94 [84.7%] episodes; ECFM, 13 [21.3%] episodes; *P* < 0.001). Patients with VRE BSI had a longer mean hospital stay prior to the BSI diagnosis (VRE, 26.3 [SD 31.5] days; ECFM, 19.4 [SD 20.5] days; *P* = 0.01). The baseline characteristics are summarized in Table S1.

### Antibiotic treatment

In total, 240 episodes (60.6%) had received antimicrobial treatment within three months before the BSI diagnosis (VRE, 120 [61.2%]; ECFM, 120 [60.0%]). The mean (SD) duration of antimicrobial treatment before the BSI diagnosis was 16.9 (20.4) days (VRE, 18 [22.3] days; ECFM: 16.1 [18.7] days).

Eighty-nine episodes (22.9%) in both groups were treated with vancomycin before the BSI diagnosis. Vancomycin treatment was initiated at a mean (SD) of 22.4 (23.3) days before VRE BSI diagnosis and 25.5 (25.7) days before ECFM BSI diagnosis. Fifty-seven episodes (14.7%) were treated with cephalosporins, 224 (57.7%) with acyl-aminopenicillins, 68 (17.5%) with quinolones, 165 (42.5%) with carbapenems, 63 (16.2%) with linezolid, and 137 (35.5%) with an antimicrobial other than those mentioned above before the BSI diagnosis. The total number exceeds 100%, as some patients were treated with several antibiotics. Compared to episodes with ECFM BSI, those with VRE BSI were significantly more likely to have received vancomycin treatment before the BSI diagnosis (VRE: 56 episodes, 29.8%; ECFM: 33 episodes, 16.5%; *P* < 0.001), and episodes with VRE BSI were also significantly more likely to have received linezolid treatment before the BSI diagnosis (VRE, 35 episodes, 18.6%; ECFM, 28 episodes, 14.0%; *P* = 0.006). All baseline characteristics are shown in Table S1.

### Risk factors for VRE BSI in the unadjusted analysis

**Fig 3:**
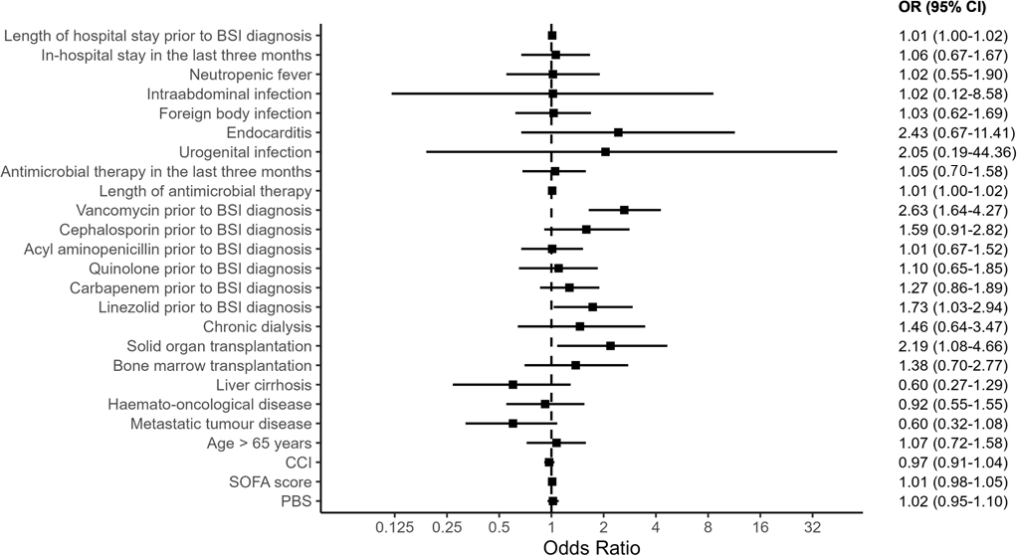
Forest plot of risk factors for VRE BSI versus ECFM BSI in the unadjusted logistic regression analysis. BSI, bloodstream infection; CCI, Charlson Comorbidity Index; CI, confidence interval; ECFM, *Enterococcus faecium*; OR, odds ratio; PBS, Pitt Bacteremia Score; SOFA, Sequential Organ Failure Assessment; VRE, vancomycin-resistant *Enterococcus faecium*.

In the unadjusted analysis (Table 1), treatment with vancomycin (OR: 2.63, 95% CI: 1.64–4.27, *P* < 0.001) and linezolid (OR: 1.73, 95% CI: 1.03–2.94, *P* = 0.041) before the BSI diagnosis was associated with VRE BSI. The risk of VRE BSI did not differ significantly according to whether patients had received prior treatment with cephalosporins (OR: 1.59, 95% CI: 0.91–2.82, *P* = 0.104), acyl-aminopenicillins (OR: 1.01, 95% CI: 0.67–1.52, *P* = 0.962), quinolones (OR: 1.10, 95% CI: 0.65–1.85, *P* = 0.721), or carbapenems (OR: 1.27, 95% CI: 0.86–1.89, *P* = 0.233). The risk of VRE BSI also did not differ significantly according to whether patients had received antimicrobial treatment in the past three months (OR: 1.05, 95% CI: 0.70–1.58, *P* = 0.803) or the duration of antimicrobial treatment before BSI diagnosis (OR: 1.01, 95% CI: 1.00–1.02, *P* = 0.062). Further, the risk of VRE BSI did not differ significantly according to the severity of underlying diseases as measured by the CCI (OR: 0.97, 95% CI: 0.91–1.04, *P* = 0.358), SOFA score (OR: 1.01, 95% CI: 0.98–1.05, *P* = 0.462), and PBS (OR: 1.02, 95% CI: 0.95–1.10, *P* = 0.587). The risk of VRE BSI was significantly higher in patients who had undergone solid organ transplantation (OR: 2.19, 95% CI: 1.08–4.65, *P* = 0.034); however, the risk of VRE was not altered significantly by metastatic cancer (OR: 0.60, 95% CI: 0.32–1.08, *P* = 0.09), hematological malignancy (OR: 0.92, 95% CI: 0.55–1.55, *P* = 0.76), liver cirrhosis (OR: 0.60, 95% CI: 0.27–1.29, *P* = 0.20), bone marrow transplantation (OR: 1.38, 95% CI: 0.70–2.77, *P* = 0.355), or chronic renal disease with dialysis (OR: 1.46, 95% CI: 0.64–3.47, *P* = 0.374) status. Patients with longer hospital stays before BSI diagnosis had a significantly higher risk of VRE BSI (OR: 1.01, 95% CI: 1.00–1.02, *P* = 0.012; Fig. 3).

**TABLE 1:** Unadjusted and multivariable logistic regression analysis of potential risk factors for vancomycin-resistant *Enterococcus faecium* bloodstream infection^*a*^

	Unadjusted analysis		Multivariable analysis	
Predictors	OR (95% CI)	*P*	OR (95% CI)	*P*
Length of hospital stay prior to BSI diagnosis	1.01 (1.00–1.02)	0.012	1.01 (1.00–1.04)	0.113
In-hospital stay in the past three months	1.06 (0.67–1.67)	0.813	0.87 (0.47–1.59)	0.644
Neutropenic fever	1.02 (0.55–1.90)	0.942	0.89 (0.40–1.97)	0.783
Intra-abdominal infection	1.02 (0.12–8.58)	0.984	0.59 (0.05–5.83)	0.641
Foreign body infection	1.03 (0.62–1.69)	0.921	1.10 (0.64–1.90)	0.734
Endocarditis	2.43 (0.67–11.41)	0.203	1.43 (0.34–7.30)	0.635
Urogenital infection	2.05 (0.19–44.36)	0.559	4.60 (0.36–110.30)	0.247
Antimicrobial therapy in the past three months	1.05 (0.70–1.58)	0.803	1.28 (0.74–2.21)	0.384
Length of antimicrobial therapy	1.01 (1.00–1.02)	0.062	0.98 (0.96–1.01)	0.163
Vancomycin prior to BSI diagnosis	2.63 (1.64–4.27)	<0.001	3.24 (1.79–6.01)	<0.001
Cephalosporin prior to BSI diagnosis	1.59 (0.91–2.82)	0.104	1.76 (0.94–3.36)	0.081
Acyl aminopenicillin prior to BSI diagnosis	1.01 (0.67–1.52)	0.962	0.93 (0.58–1.51)	0.778
Quinolone prior to BSI diagnosis	1.10 (0.65–1.85)	0.721	1.04 (0.56–1.91)	0.905
Carbapenem prior to BSI diagnosis	1.27 (0.86–1.89)	0.233	0.80 (0.46–1.37)	0.412
Linezolid prior to BSI diagnosis	1.73 (1.03–2.94)	0.041	1.66 (0.84–3.33)	0.145
Chronic dialysis	1.46 (0.64–3.47)	0.374	0.97 (0.37–2.57)	0.944
Solid organ transplantation	2.19 (1.08–4.65)	0.034	2.48 (1.09–5.90)	0.034
Bone marrow transplantation	1.38 (0.70–2.77)	0.355	1.29 (0.52–3.23)	0.584
Liver cirrhosis	0.60 (0.27–1.29)	0.200	0.49 (0.19–1.18)	0.116
Haematological malignancy	0.92 (0.55–1.55)	0.760	0.83 (0.47–1.47)	0.523
Metastatic cancer	0.60 (0.32–1.08)	0.090	0.54 (0.19–1.55)	0.257
Age >65 years	1.07 (0.72–1.58)	0.751	1.43 (0.82–2.52)	0.209
CCI	0.97 (0.91–1.04)	0.358	1.05 (0.93–1.19)	0.422
SOFA score	1.01 (0.98–1.05)	0.462	1.02 (0.96–1.08)	0.543
PBS	1.02 (0.95–1.10)	0.587	0.94 (0.83–1.07)	0.361

^*a*^ BSI, bloodstream infection; CCI, Charlson Comorbidity Index; CI, confidence interval; OR, odds ratio; PBS, Pitt Bacteremia Score; SOFA, Sequential Organ Failure Assessment.

### Risk factors for VRE BSI in the multivariable analysis

In the multivariable analysis (Table 1), treatment with vancomycin before BSI (OR: 3.24, 95% CI: 1.79–6.01, *P* < 0.001) remained an independent risk factor for VRE BSI. As in the unadjusted analysis, the risk of VRE BSI did not differ significantly according to whether patients had received prior treatment with cephalosporins, acyl-aminopenicillins, quinolones, or carbapenems. In contrast to the unadjusted analysis, the risk of VRE BSI was not significantly associated with prior linezolid use in the multivariable analysis (OR: 1.66, 95% CI: 0.84–3.33, *P* = 0.145). Further, the risk of VRE BSI did not differ significantly according to disease severity measured by the CCI, SOFA score, and PBS. As in the unadjusted analysis, solid organ transplantation was associated with a higher risk of VRE BSI (OR: 2.48, 95% CI: 1.09–5.90, *P* = 0.034), whereas the length of hospital stay before BSI diagnosis was not an independent risk factor for VRE BSI (OR: 1.01, 95% CI: 1.00–1.04, *P* = 0.113).

## DISCUSSION

Invasive VRE infections occur predominantly in healthcare settings, and identifying the risk factors for vancomycin resistance in ECFM BSI is crucial for patient outcomes. Previous studies have shown that BSIs due to VRE are more likely to occur in patients with severe underlying diseases and comorbidities (6, 8, 13, 14). Most previous studies on risk factors for VRE BSIs have evaluated individual patient factors, such as central lines and ICU admission. López-Luis et al. (15) found that a history of invasive device use (central venous catheter and endotracheal intubation) was associated with an increased risk of VRE BSI. Johnstone et al. (14) evaluated bone marrow and organ transplantation, cancer, and ICU admission as risk factors for VRE BSI. Peel et al. (6) reported that central venous catheter, neutropenia, and allogeneic bone marrow transplantation were associated with VRE BSI. In this study, solid organ transplantation was the only comorbidity identified as an independent risk factor for VRE BSI. However, the use of disease severity scores, as applied in this study, is more appropriate for determining acute disease severity, as it includes numerous variables, such as blood results and vital signs.

Only a few studies of VRE BSI have systematically included disease severity scores in the analysis. To our knowledge, this is the first study to assess whether patients with VRE BSI are generally sicker than patients with BSI due to ECFM using five disease severity scores (SAPS, SOFA, PBS, APACHE II, and CCI). Kramer et al. (11, 16) and Cheah et al. (11, 16) found no difference in the CCI between patients with VRE and those with ECFM BSI. Shay et al. (7, 13) and Lucas et al. (7, 13) found slightly higher APACHE II scores in patients with VRE BSI than in those with ECFM BSI. The results of this study indicate that patients with VRE BSI are not more severely ill than those with ECFM BSI, as determined by the five established disease severity scores for acute and chronic illness. This finding suggests that disease severity is not a reliable indicator of vancomycin resistance in patients with enterococcal BSI. Consistent with this, our group has recently shown that ECFM BSI is a risk factor for mortality, regardless of the presence of vancomycin resistance (12).

In this study, the unadjusted logistic regression analysis showed that prior treatment with vancomycin or linezolid, solid organ transplantation, and longer length of hospital stay were associated with VRE BSI. However, in the multivariable analysis, prior vancomycin treatment and solid organ transplantation were the only independent risk factors for VRE BSI. Vancomycin is a glycopeptide that inhibits cell wall synthesis in Gram-positive bacteria. Enterococci can acquire *van* genes that carry resistance mechanisms to modify the enterococcal cell wall structure, thereby reducing the affinity for vancomycin. Enterococci are natural colonizers of the human intestine, and prior treatment with vancomycin is a known risk factor for both colonization and infection with VRE (3, 5, 7, 17). This is probably because the frequent and prolonged use of vancomycin disrupts the normal gut flora and increases the selective pressure for the spread of VRE. However, the influence of prior gastrointestinal VRE colonization on the development of VRE BSI remains uncertain. Presumably, VRE colonization is not clinically relevant in most cases (18). In this study, the proportion of patients with preceding rectal VRE colonization was significantly higher in patients with VRE BSI than in those with ECFM BSI; however, the effect could not be further evaluated in the logistic regression analysis owing to missing data.

Previous studies have shown that prior cephalosporin treatment increases the risk of VRE infection and colonization (9, 19, 20). Enterococci are naturally resistant to cephalosporins, and selection pressure during cephalosporin treatment may promote gastrointestinal colonization with enterococci. In this study, prior cephalosporin treatment was not a risk factor for VRE BSI, possibly because of the low number of patients with VRE and those treated with cephalosporins.

The standard of care diagnostic tool for diagnosing BSI is blood cultures with the cultivation of microorganisms and subsequent susceptibility testing. The disadvantage of culture-based methods is that no molecular data on resistance genes are collected, and those methods take around 48–72 hours to yield results, which delays treatment and infection control measures. In contrast, rapid molecular diagnostic platforms, such as PCR, have shown promising results in the early detection of VRE, especially in screening settings. In contrast, PCR for diagnosing sepsis from whole blood is not yet a diagnostic routine standard. PCR results can usually be expected in a few hours, and they can detect resistance genes for quicker clinical decision-making. Disadvantages are higher costs, lower sensitivity and specificity (especially false-positive results), and the lack of complete antimicrobial resistance patterns compared to standard of care, which only allows genotypic resistance detection of previously known genes (21–23). In this study, cultivation was applied as a standard of care, and no information on molecular data were collected, which is a limitation. Future research should evaluate the impact of rapid molecular diagnostic platforms on clinical outcomes and antimicrobial stewardship measures.

This study has some further limitations. These include its retrospective design with a high number of missing data and the long data collection period (from 2010 to 2019) owing to the low incidence of VRE in Munich. Additionally, no statistical multiple testing comparison was conducted. There were only a few significant correlations compared to the number of potential predictors analyzed, which limits the significance of the results. Prospective studies should be considered to confirm the findings of this study. In addition, expanding the study to include a more diverse range of healthcare settings and patient demographics would enhance the generalizability of the results.

In conclusion, this study suggests that prior vancomycin treatment and solid organ transplantation are independent risk factors for vancomycin resistance in patients with ECFM BSI. However, none of the five widely used disease severity scores (SAPS, SOFA, PBS, APACHE-II, and CCI) was associated with VRE BSI, suggesting that disease severity is not a reliable predictor of vancomycin resistance.

The results of this study indicate that patients with suspected or confirmed ECFM BSI should be screened for prior vancomycin therapy. Considering these risk factors might improve empirical therapy and should not be limited to critically ill patients, given that disease severity is not predictive of VRE-BSI.

## Data Availability

The data sets used or analyzed during the current study are available from the corresponding author on reasonable request.

## SUPPLEMENTAL MATERIAL

Table S1 spectrum.00052-25-s0001.docx Baseline characteristics of patients with vancomycin-resistant *Enterococcus faecium* and *Enterococcus faecium* bloodstream infection.

ASM does not own the copyrights to Supplemental Material that may be linked to, or accessed through, an article. The authors have granted ASM a non-exclusive, world-wide license to publish the Supplemental Material files. Please contact the corresponding author directly for reuse.

## References

[1] Control ECfDPa. 2023. Antimicrobial resistance surveillance in Europe 2023–2021 data

[2] Fiore E, Van Tyne D, Gilmore MS. 2019. Pathogenicity of enterococci. Microbiol Spectr 7. doi:10.1128/microbiolspec.GPP3-0053-2018 PMC6629438 31298205

[3] Zaas AK, Song X, Tucker P, Perl TM. 2002. Risk factors for development of vancomycin-resistant enterococcal bloodstream infection in patients with cancer who are colonized with vancomycin-resistant enterococci. Clin Infect Dis 35:1139–1146. doi:10.1086/342904 12410472

[4] Ford CD, Lopansri BK, Haydoura S, Snow G, Dascomb KK, Asch J, Bo Petersen F, Burke JP. 2015. Frequency, risk factors, and outcomes of vancomycin-resistant Enterococcus colonization and infection in patients with newly diagnosed acute leukemia: different patterns in patients with acute myelogenous and acute lymphoblastic leukemia. Infect Control Hosp Epidemiol 36:47–53. doi:10.1017/ice.2014.3 25627761

[5] Worth LJ, Thursky KA, Seymour JF, Slavin MA. 2007. Vancomycin-resistant Enterococcus faecium infection in patients with hematologic malignancy: patients with acute myeloid leukemia are at high-risk. Eur J Haematol 79:226–233. doi:10.1111/j.1600-0609.2007.00911.x 17655696

[6] Peel T, Cheng AC, Spelman T, Huysmans M, Spelman D. 2012. Differing risk factors for vancomycin-resistant and vancomycin-sensitive enterococcal bacteraemia. Clin Microbiol Infect 18:388–394. doi:10.1111/j.1469-0691.2011.03591.x 21848977

[7] Shay DK, Maloney SA, Montecalvo M, Banerjee S, Wormser GP, Arduino MJ, Bland LA, Jarvis WR. 1995. Epidemiology and mortality risk of vancomycin-resistant enterococcal bloodstream infections. J Infect Dis 172:993–1000. doi:10.1093/infdis/172.4.993 7561221

[8] Bhavnani SM, Drake JA, Forrest A, Deinhart JA, Jones RN, Biedenbach DJ, Ballow CH. 2000. A nationwide, multicenter, case-control study comparing risk factors, treatment, and outcome for vancomycin-resistant and -susceptible enterococcal bacteremia. Diagn Microbiol Infect Dis 36:145–158. doi:10.1016/s0732-8893(99)00136-4 10729656

[9] Stagliano DR, Susi A, Adams DJ, Nylund CM. 2021. Epidemiology and outcomes of vancomycin-resistant enterococcus infections in the U.S. military health system. Mil Med 186:100–107. doi:10.1093/milmed/usaa229 33499465

[10] Ong DSY, Bonten MJM, Safdari K, Spitoni C, Frencken JF, Witteveen E, Horn J, Klein Klouwenberg PMC, Cremer OL, MARS consortium. 2015. Epidemiology, management, and risk-adjusted mortality of ICU-acquired enterococcal bacteremia. Clin Infect Dis 61:1413–1420. doi:10.1093/cid/civ560 26179013

[11] Cheah ALY, Spelman T, Liew D, Peel T, Howden BP, Spelman D, Grayson ML, Nation RL, Kong DCM. 2013. Enterococcal bacteraemia: factors influencing mortality, length of stay and costs of hospitalization. Clin Microbiol Infect 19:E181–E189. doi:10.1111/1469-0691.12132 23398607

[12] Rothe K, Bachfischer T, Karapetyan S, Hapfelmeier A, Wurst M, Gleich S, Dichtl K, Schmid RM, Triebelhorn J, Wagner L, Erber J, Voit F, Burgkart R, Obermeier A, Seibold U, Busch DH, Rämer PC, Spinner CD, Schneider J. 2023. Are enterococcal bloodstream infections an independent risk factor for a poorer 5-year survival or just a marker for severity of illness?-The Munich multicentric enterococci cohort. Microbiol Spectr 11:e0258523. doi:10.1128/spectrum.02585-23 37791770 PMC10715215

[13] Lucas GM, Lechtzin N, Puryear DW, Yau LL, Flexner CW, Moore RD. 1998. Vancomycin-resistant and vancomycin-susceptible enterococcal bacteremia: comparison of clinical features and outcomes. Clin Infect Dis 26:1127–1133. doi:10.1086/520311 9597241

[14] Johnstone J, Chen C, Rosella L, Adomako K, Policarpio ME, Lam F, Prematunge C, Garber G, Ontario VRE Investigators. 2018. Patient- and hospital-level predictors of vancomycin-resistant Enterococcus (VRE) bacteremia in Ontario, Canada. Am J Infect Control 46:1266–1271. doi:10.1016/j.ajic.2018.05.003 29903421

[15] López-Luis BA, Sifuentes-Osornio J, Lambraño-Castillo D, Ortiz-Brizuela E, Ramírez-Fontes A, Tovar-Calderón YE, Leal-Vega FJ, Bobadilla-Del-Valle M, Ponce-de-León A. 2021. Risk factors and outcomes associated with vancomycin-resistant Enterococcus faecium and ampicillin-resistant Enterococcus faecalis bacteraemia: a 10-year study in a tertiary-care centre in Mexico City. J Glob Antimicrob Resist 24:198–204. doi:10.1016/j.jgar.2020.12.005 33359937

[16] Kramer TS, Remschmidt C, Werner S, Behnke M, Schwab F, Werner G, Gastmeier P, Leistner R. 2018. The importance of adjusting for enterococcus species when assessing the burden of vancomycin resistance: a cohort study including over 1000 cases of enterococcal bloodstream infections. Antimicrob Resist Infect Control 7:133. doi:10.1186/s13756-018-0419-9 30459945 PMC6234683

[17] Zacharioudakis IM, Zervou FN, Ziakas PD, Rice LB, Mylonakis E. 2015. Vancomycin-resistant enterococci colonization among dialysis patients: a meta-analysis of prevalence, risk factors, and significance. Am J Kidney Dis 65:88–97. doi:10.1053/j.ajkd.2014.05.016 25042816

[18] Mutters NT, Mersch-Sundermann V, Mutters R, Brandt C, Schneider-Brachert W, Frank U. 2013. Control of the spread of vancomycin-resistant enterococci in hospitals. Dtsch Ärztebl Int 110:725–731. doi:10.3238/arztebl.2013.0725 24222791 PMC3822708

[19] Yan MY, He YH, Ruan GJ, Xue F, Zheng B, Lv Y. 2023. The prevalence and molecular epidemiology of vancomycin-resistant Enterococcus (VRE) carriage in patients admitted to intensive care units in Beijing, China. J Microbiol Immunol Infect 56:351–357. doi:10.1016/j.jmii.2022.07.001 35922268

[20] Janjusevic A, Cirkovic I, Minic R, Stevanovic G, Soldatovic I, Mihaljevic B, Vidovic A, Markovic Denic L. 2022. Predictors of vancomycin-resistant Enterococcus spp. intestinal carriage among high-risk patients in University Hospitals in Serbia. Antibiotics (Basel) 11:1228. doi:10.3390/antibiotics11091228 36140006 PMC9495008

[21] Holzknecht BJ, Hansen DS, Nielsen L, Kailow A, Jarløv JO. 2017. Screening for vancomycin-resistant enterococci with Xpert® vanA/vanB: diagnostic accuracy and impact on infection control decision making. New Microbes New Infect 16:54–59. doi:10.1016/j.nmni.2016.12.020 28203378 PMC5295639

[22] Marner ES, Wolk DM, Carr J, Hewitt C, Dominguez LL, Kovacs T, Johnson DR, Hayden RT. 2011. Diagnostic accuracy of the Cepheid GeneXpert vanA/vanB assay ver. 1.0 to detect the vanA and vanB vancomycin resistance genes in Enterococcus from perianal specimens. Diagn Microbiol Infect Dis 69:382–389. doi:10.1016/j.diagmicrobio.2010.11.005 21396533

[23] Werner G, Serr A, Schütt S, Schneider C, Klare I, Witte W, Wendt C. 2011. Comparison of direct cultivation on a selective solid medium, polymerase chain reaction from an enrichment broth, and the BD GeneOhm VanR Assay for identification of vancomycin-resistant enterococci in screening specimens. Diagn Microbiol Infect Dis 70:512–521. doi:10.1016/j.diagmicrobio.2011.04.004 21767707

