# Supplementary material for: Risk factors for vancomycin resistance in patients with Enterococcus faecium bloodstream infections: an analysis of the Munich Multicentric Enterococci Cohort
Source: Microbiol Spectr. 2025 Jun 5;13(7):e00052-25. doi: 10.1128/spectrum.00052-25 (PMC12210936; doi:10.1128/spectrum.00052-25)
Supplement: Table S1 — Baseline characteristics of patients with vancomycin-resistant Enterococcus faecium and Enterococcus faecium bloodstream infection. [file spectrum.00052-25-s0001.docx]

**Supplementary material**

**Supplementary Table 1: Baseline characteristics of patients with vancomycin-resistant *Enterococcus faecium* and *Enterococcus faecium* bloodstream infection**

|  | **VRE (N=196)** | **ECFM (N=200)** | **Total (N=396)** | ***P*-value** |
| --- | --- | --- | --- | --- |
| **Early rehabilitation** |  |  |  | 0.184 |
| No | 195 (99.5%) | 196 (98.0%) | 391 (98.7%) |  |
| Yes | 1 (0.5%) | 4 (2.0%) | 5 (1.3%) |  |
| **ICU** |  |  |  | 0.952 |
| No | 118 (60.2%) | 121 (60.5%) | 239 (60.4%) |  |
| Yes | 78 (39.8%) | 79 (39.5%) | 157 (39.6%) |  |
| **Haematological ward** |  |  |  | 0.299 |
| No | 160 (81.6%) | 171 (85.5%) | 331 (83.6%) |  |
| Yes | 36 (18.4%) | 29 (14.5%) | 65 (16.4%) |  |
| **Nephrology ward** |  |  |  | 0.418 |
| No | 191 (97.4%) | 192 (96.0%) | 383 (96.7%) |  |
| Yes | 5 (2.6%) | 8 (4.0%) | 13 (3.3%) |  |
| **PBS** |  |  |  | 0.643 |
| Missing | 76 | 52 | 128 |  |
| Mean (SD) | 3.3 (2.8) | 3.2 (2.9) | 3.3 (2.9) |  |
| **Neutropenic fever** |  |  |  | 0.942 |
| No | 173 (88.3%) | 177 (88.5%) | 350 (88.4%) |  |
| Yes | 23 (11.7%) | 23 (11.5%) | 46 (11.6%) |  |
| **Intra-abdominal infection** |  |  |  | 0.984 |
| No | 194 (99.0%) | 198 (99.0%) | 392 (99.0%) |  |
| Yes | 2 (1.0%) | 2 (1.0%) | 4 (1.0%) |  |
| **Foreign body infection** |  |  |  | 0.921 |
| No | 157 (80.1%) | 161 (80.5%) | 318 (80.3%) |  |
| Yes | 39 (19.9%) | 39 (19.5%) | 78 (19.7%) |  |
| **Endocarditis** |  |  |  | 0.189 |
| No | 189 (96.4%) | 197 (98.5%) | 386 (97.5%) |  |
| Yes | 7 (3.6%) | 3 (1.5%) | 10 (2.5%) |  |
| **Urogenital infection** |  |  |  | 0.550 |
| No | 194 (99.0%) | 199 (99.5%) | 393 (99.2%) |  |
| Yes | 2 (1.0%) | 1 (0.5%) | 3 (0.8%) |  |
| **Rectal VRE colonisation** |  |  |  | **< 0.001** |
| Missing | 85 | 139 | 224 |  |
| Negative | 17 (15.3%) | 48 (78.7%) | 65 (37.8%) |  |
| Positive | 94 (84.7%) | 13 (21.3%) | 107 (62.2%) |  |
| **Rectal VRE screening prior to BSI diagnosis** |  |  |  | **0.008** |
| Missing | 85 | 139 | 224 |  |
| No | 54 (48.6%) | 17 (27.9%) | 71 (41.3%) |  |
| Yes | 57 (51.4%) | 44 (72.1%) | 101 (58.7%) |  |
| **Antibiotic treatment in the past 3 months** |  |  |  | 0.803 |
| No | 76 (38.8%) | 80 (40.0%) | 156 (39.4%) |  |
| Yes | 120 (61.2%) | 120 (60.0%) | 240 (60.6%) |  |
| **Length of hospital stay until BSI diagnosis (days)** |  |  |  | **0.010** |
| Mean (SD) | 26.3 (31.5) | 19.4 (20.5) | 22.8 (26.7) |  |
| **Length of hospitalisation until BSI diagnosis > 5 days** |  |  |  | **0.046** |
| No | 39 (19.9%) | 57 (28.5%) | 96 (24.2%) |  |
| Yes | 157 (80.1%) | 143 (71.5%) | 300 (75.8%) |  |
| **Duration of antibiotic therapy (days) prior to BSI diagnosis** |  |  |  | 0.384 |
| Missing | 35 | 11 | 46 |  |
| Mean (SD) | 18.0 (22.3) | 16.1 (18.7) | 16.9 (20.4) |  |
| **Vancomycin prior to BSI diagnosis** |  |  |  | **< 0.001** |
| Missing | 8 | 0 | 8 |  |
| No | 123 (65.4%) | 165 (82.5%) | 288 (74.2%) |  |
| Yes | 56 (29.8%) | 33 (16.5%) | 89 (22.9%) |  |
| Yes, but unsure if prior to BSI diagnosis | 9 (4.8%) | 2 (1.0%) | 11 (2.8%) |  |
| **Cephalosporin prior to BSI diagnosis** |  |  |  | 0.188 |
| Missing | 8 | 0 | 8 |  |
| No | 151 (80.3%) | 174 (87.0%) | 325 (83.8%) |  |
| Yes | 33 (17.6%) | 24 (12.0%) | 57 (14.7%) |  |
| Yes, but unsure if prior to BSI diagnosis | 4 (2.1%) | 2 (1.0%) | 6 (1.5%) |  |
| **Antibiotics from aminopenicillin group prior to BSI diagnosis** |  |  |  | 0.259 |
| Missing | 8 | 0 | 8 |  |
| No | 75 (39.9%) | 76 (38.0%) | 151 (38.9%) |  |
| Yes | 104 (55.3%) | 120 (60.0%) | 224 (57.7%) |  |
| Yes, but unsure if prior to BSI diagnosis | 9 (4.8%) | 4 (2.0%) | 13 (3.4%) |  |
| **Fluoroquinolone prior to BSI diagnosis** |  |  |  | 0.127 |
| Missing | 8 | 0 | 8 |  |
| No | 148 (78.7%) | 165 (82.5%) | 313 (80.7%) |  |
| Yes | 34 (18.1%) | 34 (17.0%) | 68 (17.5%) |  |
| Yes, but unsure if prior to BSI diagnosis | 6 (3.2%) | 1 (0.5%) | 7 (1.8%) |  |
| **Carbapenem prior to BSI diagnosis** |  |  |  | **0.034** |
| Missing | 8 | 0 | 8 |  |
| No | 94 (50.0%) | 111 (55.5%) | 205 (52.8%) |  |
| Yes | 80 (42.6%) | 85 (42.5%) | 165 (42.5%) |  |
| Yes, but unsure if prior to BSI diagnosis | 14 (7.4%) | 4 (2.0%) | 18 (4.6%) |  |
| **Linezolid prior to BSI** |  |  |  | **0.006** |
| Missing | 8 | 0 | 8 |  |
| No | 141 (75.0%) | 170 (85.0%) | 311 (80.2%) |  |
| Yes | 35 (18.6%) | 28 (14.0%) | 63 (16.2%) |  |
| Yes, but unsure if prior to BSI diagnosis | 12 (6.4%) | 2 (1.0%) | 14 (3.6%) |  |
| **Other antibiotics prior to BSI diagnosis** |  |  |  | 0.157 |
| Missing | 8 | 0 | 8 |  |
| No | 107 (56.9%) | 122 (61.0%) | 229 (59.0%) |  |
| Yes | 66 (35.1%) | 71 (35.5%) | 137 (35.3%) |  |
| Yes, but unsure if prior to BSI diagnosis | 15 (8.0%) | 7 (3.5%) | 22 (5.7%) |  |
| **CCI** |  |  |  | 0.359 |
| Mean (SD) | 5.1 (2.9) | 5.4 (3.1) | 5.3 (3.0) |  |
| **APACHE II score** |  |  |  | 0.576 |
| Missing | 102 | 79 | 181 |  |
| Mean (SD) | 21.9 (8.5) | 21.3 (8.7) | 21.6 (8.6) |  |
| **APACHE II score > 8** |  |  |  | 0.521 |
| Missing | 102 | 79 | 181 |  |
| No | 3 (3.2%) | 6 (5.0%) | 9 (4.2%) |  |
| Yes | 91 (96.8%) | 115 (95.0%) | 206 (95.8%) |  |
| **SOFA score** |  |  |  | 0.998 |
| Missing | 65 | 64 | 129 |  |
| Mean (SD) | 6.8 (5.8) | 6.8 (5.9) | 6.8 (5.9) |  |
| **SAPS score** |  |  |  | 0.275 |
| Missing | 102 | 103 | 205 |  |
| Mean (SD) | 44.8 (13.9) | 47.1 (14.9) | 45.9 (14.5) |  |
| **Nosocomial infection** |  |  |  | 0.132 |
| No | 28 (14.3%) | 40 (20.0%) | 68 (17.2%) |  |
| Yes | 168 (85.7%) | 160 (80.0%) | 328 (82.8%) |  |
| **Hospital stay in the past 3 months** |  |  |  | 0.813 |
| No | 47 (24.0%) | 50 (25.0%) | 97 (24.5%) |  |
| Yes | 149 (76.0%) | 150 (75.0%) | 299 (75.5%) |  |
| **Type of admission** |  |  |  | 0.434 |
| From home | 127 (64.8%) | 137 (68.5%) | 264 (66.7%) |  |
| From nursing home/hospital/rehabilitation | 69 (35.2%) | 63 (31.5%) | 132 (33.3%) |  |
| **CCI ≥5** |  |  |  | 0.699 |
| No | 92 (46.9%) | 90 (45.0%) | 182 (46.0%) |  |
| Yes | 104 (53.1%) | 110 (55.0%) | 214 (54.0%) |  |
| **Dialysis** |  |  |  | 0.372 |
| No | 182 (92.9%) | 190 (95.0%) | 372 (93.9%) |  |
| Yes | 14 (7.1%) | 10 (5.0%) | 24 (6.1%) |  |
| **Solid organ transplantation** |  |  |  | **0.031** |
| No | 172 (87.8%) | 188 (94.0%) | 360 (90.9%) |  |
| Yes | 24 (12.2%) | 12 (6.0%) | 36 (9.1%) |  |
| **Bone marrow transplantation** |  |  |  | 0.353 |
| No | 175 (89.3%) | 184 (92.0%) | 359 (90.7%) |  |
| Yes | 21 (10.7%) | 16 (8.0%) | 37 (9.3%) |  |
| **Liver cirrhosis** |  |  |  | 0.196 |
| No | 185 (94.4%) | 182 (91.0%) | 367 (92.7%) |  |
| Yes | 11 (5.6%) | 18 (9.0%) | 29 (7.3%) |  |
| **Haematological malignancy** |  |  |  | 0.666 |
| Missing | 0 | 22 | 22 |  |
| No | 163 (83.2%) | 145 (81.5%) | 308 (82.4%) |  |
| Yes | 33 (16.8%) | 33 (18.5%) | 66 (17.6%) |  |
| **Metastatic cancer** |  |  |  | 0.088 |
| No | 176 (89.8%) | 168 (84.0%) | 344 (86.9%) |  |
| Yes | 20 (10.2%) | 32 (16.0%) | 52 (13.1%) |  |
| **Age > 65 years** |  |  |  | 0.751 |
| No | 89 (45.4%) | 94 (47.0%) | 183 (46.2%) |  |
| Yes | 107 (54.6%) | 106 (53.0%) | 213 (53.8%) |  |

APACHE II, Acute Physiology and Chronic Health Evaluation; BSI, bloodstream infection; CCI, Charlson Comorbidity Index; ECFM, *Enterococcus faecium*; ICU, intensive care unit; PBS, Pitt Bacteremia Score; SD, standard deviation; SOFA, Sequential Organ Failure Assessment; SAPS; Simplified Acute Physiology Score; VRE, vancomycin-resistant *Enterococcus faecium*.
